# Mechanism of Photocurrent Degradation and Contactless Healing in *p*-Type Mg-Doped Gallium Nitride Thin Films

**DOI:** 10.3390/nano12060899

**Published:** 2022-03-09

**Authors:** Xiaoyan Wu, Wei Li, Qingrong Chen, Caixia Xu, Jiamian Wang, Lingyuan Wu, Guodong Liu, Weiping Wang, Ting Li, Ping Chen, Long Xu

**Affiliations:** 1 Institute of Fluid Physics, China Academy of Engineering Physics, Mianyang 621900, China; wuxiaoyan1219@sina.cn (X.W.); vleefoxtrot@outlook.com (W.L.); guodliu@126.com (G.L.); wwpwzc@yeah.net (W.W.); 2 Chongqing Key Laboratory of Micro&Nano Structure Optoelectronics, School of Physical Science and Technology, Southwest University, Chongqing 400715, China; cqr26704034132022@163.com (Q.C.); longhowl_harold@126.com (J.W.); xiaotuzi2019@office365.swu.edu.cn (T.L.); pingchen@swu.edu.cn (P.C.); 3 School of Primary Education, Chongqing Normal University, Chongqing 400700, China

**Keywords:** Mg-doped gallium nitride, light-induced degradation, photocurrent device, contactless healing

## Abstract

Light-induced degradation (LID) phenomenon is commonly found in optoelectronics devices. Self-healing effect in halide lead perovskite solar cells was investigated since the electrons and holes in the shallow traps could escape easily at room temperature. However, the degradation in the semiconductors could not easily recover at room temperature, and many of them needed annealing at temperatures in the several hundreds, which was not friendly to the integrated optoelectronic semiconductor devices. To solve this problem, in this work, LID effect of photocurrent in p-type Mg-doped gallium nitride thin films was investigated, and deep defect and vacancy traps played a vital role in the LID and healing process. This work provides a contactless way to heal the photocurrent behavior to its initial level, which is desirable in integrated devices.

## 1. Introduction

Light-induced degradation (LID) phenomenon has been widely observed in solar cells in silicon and halide lead perovskites-based devices and light emitting diodes in metal oxides and metal nitride semiconductor materials, which impedes their application in high performance electrooptical and electronics devices [1,2,3,4,5]. The self-healing effect in halide lead perovskite solar cells was investigated, and the defects that assisted shallow electron traps played a vital role in the thermal healing process [6,7]. Silicon-based passivated emitter rear contact (PERC) solar cells suffer the LID effect when they are first illuminated by the sunlight in Czochralski-grown p-type Boron doped silicon substrates, which mainly originated from the formation of the boron–oxygen complex [8,9,10]. The loss of PERC solar cells efficiency with running age was attributed primarily to both light and elevated temperature induced degradation (LeTID) and LID [11,12,13]. In recent years, similar light-induced degradation of photocurrent and refractive index was found in many wide bandgap semiconductor and single crystal materials [14,15,16,17,18]. However, the degradation in the semiconductors could not quickly recover at room temperature, and many of them needed annealing at temperatures higher than 300 °C, which was not friendly to the integrated optoelectronic semiconductor devices [19,20]. The mechanism of the degradation and healing of the photocurrent were not proposed clearly in these semiconductors.

To solve these issues, in this work, ultraviolet light-induced degradation of photocurrent in p-type Mg-doped gallium nitride thin films was investigated, grown by using metal organic chemical vapor deposition (MOCVD). A physical picture based on deep electron traps (DET) and deep hole traps (DHT) was proposed, which contributed to the photocurrent efficiency degradation. Electrons and holes in these traps could not easily escape at room temperature in DET and DHT. After the photocurrent response degraded, an infrared light source at 808 nm was used to expose the surface of the devices, and the photocurrent and free hole carrier concentration largely recovered to the original condition. This work is suitable for light-induced degradation of photocurrent devices based on defects assisted electron and hole carrier traps, including halide leads perovskites, zinc dioxide, and gallium nitrides semiconductors. We report our work, as follows.

## 2. Materials and Methods

**Figure 1:**
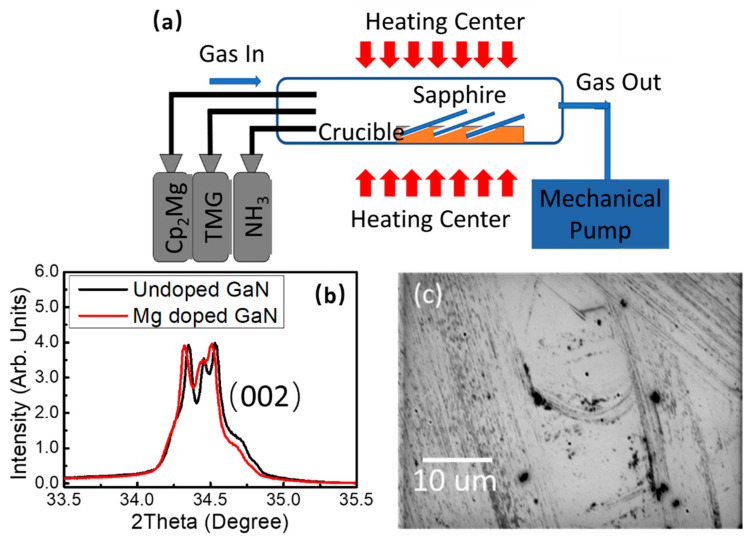
(**a**) Schematic illustration of the MOCVD system; (**b**) X-ray diffraction spectrum of the Mg-doped and undoped GaN thin film; (**c**) Scanning electron microscopy of the Mg-doped GaN thin film.

Mg-doped gallium nitride thin films were grown on c-plane sapphire (001) by using MOCVD, as seen in Figure 1a. Trimethylgallium (TMG, Strem Chemicals Inc., Newburyport, MA, USA, 99.9999%), high purity ammonia (Rising Gas Inc., Chongqing, China, 99.9995%), and bis(ethyl-cyclopentadienyl) magnesium (Cp_2_Mg, Aladdin^®^, Los Angeles, CA, USA, 99.99%) were used as the gas sources of gallium, nitrogen, and magnesium, respectively. The c-sapphire substrates were inserted into the custom-built crucible’s lots and were put into the furnace’s hot zone. As listed in Table 1, firstly, a 50 nm thick gallium nitride buffer layer was grown at 520 °C by controlling the flow rate of TMG and ammonia at 8.3 µmol/min and 0.5 sccm, respectively, under a vacuum pressure of 80 Torr. After growing the buffer layer, Cp_2_Mg gas with a flow rate of 0.4 µmol/min was added as a magnesium source to grow the Mg-doped gallium nitride active layer, and the growth temperature was raised to 850 °C. When the growth procedures were finished, the Mg-doped GaN thin films were annealed at 750 °C using a rapid thermal annealing system in nitrogen ambient.

To confirm the unit structure and surface appearance of the Mg-doped GaN thin films, X-ray diffraction spectra and the scanning electron microscope photographs were investigated. After that, the absorption and photoluminescence spectra were measured to analyze the bandgap and defects of the thin films. To explore the electrical properties of the samples, photocurrent response variation with time and bias voltage were also measured under the illumination of ultraviolet light at 365 nm and 275 nm. Photocurrent devices related parameters such as responsivity, detectivity, and external quantum efficiency were calculated. To further analyze the degradation and recovering response of the photocurrent response, the carrier concentration before and after ultraviolet light exposure was measured by using Hall measurement system. The detailed experimental types of equipment we used in this work were as follows. The surface morphology of the Mg-doped GaN thin films was investigated by using the electron scanning microscope (SEM, JEOL JSM-7100F), and the compositions of magnetism, gallium, and nitrogen were analyzed by using the energy dispersive spectrometer (Edx, JEOL JSM-7100F). The unit structure of the samples were analyzed by using an X-ray diffraction measurement system (D/MAX-rB, Rigaku, Japan). The room temperature ground state absorption spectrum was measured using a UV-Vis spectrophotometer (SHIMADZU Inc., Kyoto, Japan, UV-2600). The photoluminescence emission spectrum was excited by using a nanosecond laser centered at 354.7 nm (Shenzhen RFH Laser Technology Co. Ltd., Shenzhen, China, 355-DFNA 108-3/30), and recorded by a spectrometer (Ocean Optics Inc., Dunedin, FL, USA, QE65Pro). The carrier concentrations of the p-type Mg-doped GaN samples were measured using a Hall measurement system ((Joule Yacht Inc., Wuhan, China, HET-RT). Interdigital gold contacts with the interval of 30 µm were deposited on the thin films using UV photolithography (Chinese Academy of Sciences, URE-2000/35), E-beam evaporation (LJ-UHV Technology Inc., Taiwan, China, Model LJ-550E), and standard lift-off technology. The photocurrent properties of the samples were investigated using a two-tips probe station, and the photocurrent response and dynamics were detected by a high-resolution source meter (Keithley, Tektronix Inc., Beaverton, OR, USA, Series 2400) and a picoammeter (Keithley, Tektronix Inc., Beaverton, OR, USA, Model 6487). 

## 3. Results

As seen in Figure 1b, a typical wurtzite-type hexagonal unit structure was recorded in the X-ray diffraction spectrum, and the prominent peak centered at 34.5° corresponds to the diffraction peak of (002). When Mg^2+^ ions were doped into the GaN wurtzite hexagonal structure, the diffraction peak showed an obvious blue shift, since the ionic radius of Mg^2+^ ion was a little bit larger than that of Ga^3+^ ion. As seen in Figure 1c, the surface appearance of the Mg-doped GaN thin film was measured by using a scanning electron microscope. Many textures could be observed on the surface, since the introduction of magnesium ions tended to aggregate and caused unevenness or protrusion.

**Figure 2:**
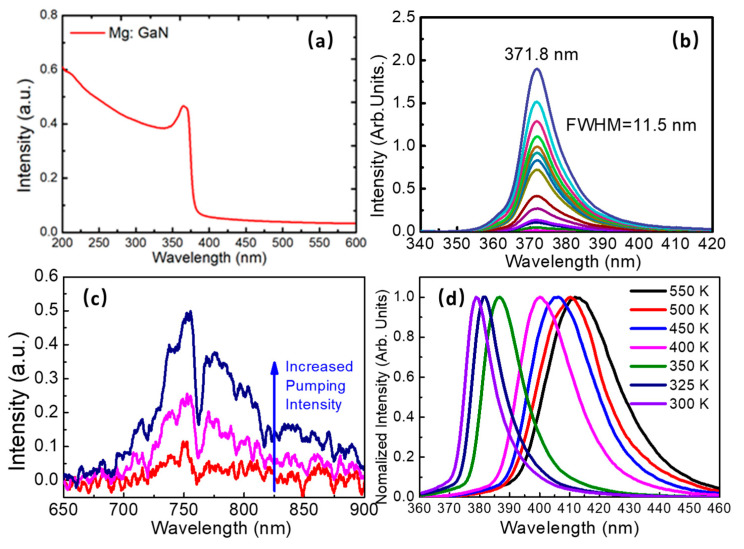
(**a**) Absorption spectrum of the Mg-doped GaN thin film; (**b**) Photoluminescence spectra of the Mg-doped GaN thin film excited by the 355 nm nanosecond laser; (**c**) Defect luminescence of the Mg-doped GaN thin film; (**d**) Temperature-dependent photoluminescence of the Mg-doped GaN thin film.

A typical direct band absorption spectrum was seen in Figure 2a, and the bandgap of the Mg-doped GaN thin film was determined as 3.41 eV by calculating the intercept of the bandgap edge with the x-axis of the direct bandgap semiconductor Tauc-plot absorption curve. The interband photoluminescence emission spectra centered at 371.8 nm were shown in Figure 2b with a full width at half maximum (FWMH) of 11.6 nm. A broadband defect photoluminescence emission could be observed from 700 nm to 900 nm, as seen in Figure 2c, which corresponds to the radiative transition from deep donors to deep vacancies related centers (both V_Ga_ and V_N_-Mg_Ga_) [21,22]. The spectral ups and downs are due to the fact that the defective energy level luminescence was too weak to fully deduct ambient noise when collecting the spectrum, but it did not influence the defect luminescence emission center and range. To further study the photoluminescence property of Mg-doped GaN thin films, temperature-dependent photoluminescence spectra were measured with increased temperature, as seen in Figure 2d. The emission peaks showed a wide range redshift from 371.5 nm to 411.9 nm, with broadened FWMH from 11.6 nm to 27.8 nm at 500 K. It provides a convenient way to modulate the wavelength of the luminescence over a wide range.

**Figure 3:**
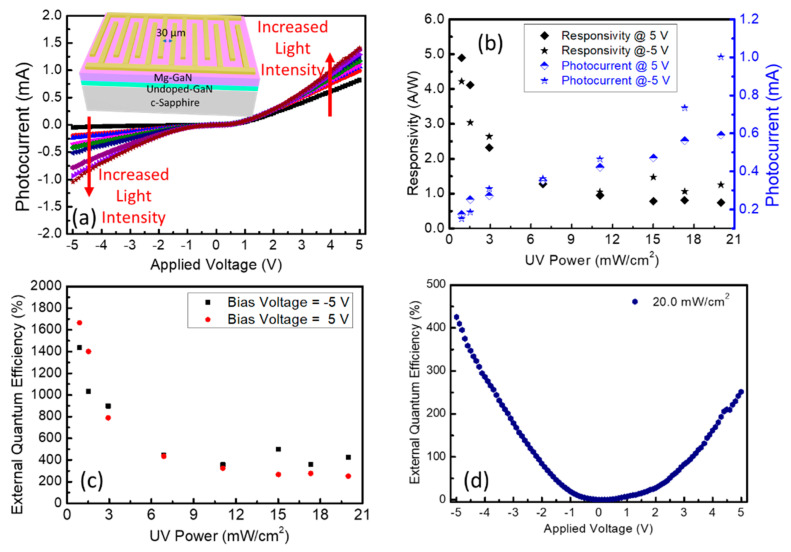
(**a**) Typical Schottky type I–V characteristic curve with and without UV light illumination of Mg–doped GaN thin film with Au interdigital contact on the top (the inset is the schematic diagram of the interdigital metal contact structure); (**b**) Responsivity and photocurrent intensity of the Mg–doped GaN device versus UV light power; (**c**) External quantum efficiency of the Mg–doped GaN device versus UV light power; (**d**) External quantum efficiency of the Mg–doped GaN device versus applied bias voltage.

To further study the electrical properties of the Mg-doped GaN thin films, as seen in the inset of Figure 3a, interdigital metal contact was deposited on the thin films with Au (100 nm) by using the E-beam evaporation system. As shown in the black curve of Figure 3a, a typical Schottky type I-V characteristic curve was measured with a threshold of approximately 2 V by using the probe station, which also implied an excellent p-type conductivity of the sample. Under the illumination of the ultraviolet (UV) light at 365 nm, as seen in Figure 3b, high responsivity up to 4.7 A/W was calculated based on the equation of $R_{\lambda }=\frac{I_{\lambda }-I_{d}}{P_{\lambda }\;S}$ at the pumping intensity of 0.89 mW/cm^2^ and decreased as the pumping intensity increased, where I_λ_ is the photocurrent, I_d_ is the dark current, P_λ_ is the pumping intensity, and *S* is the effective illuminative area on the device. The photocurrent also increased with the power of UV light. As shown in Figure 3c, an external quantum efficiency (EQE) of 1600% at the UV intensity of 0.89 mW/cm^2^ with an applied voltage of 5V was obtained by using the equation of $\mathrm{EQE}=\frac{{\mathrm{hcR}}_{\lambda }}{e\lambda }$, where h is plank constant, c is the speed of light in the vacuum, and λ is the wavelength of the pumping light. The EQE of the photocurrent devices still kept 200% or more at higher UV light intensity, and it changed with the applied voltage on the devices, as exhibited in Figure 3d.

**Figure 4:**
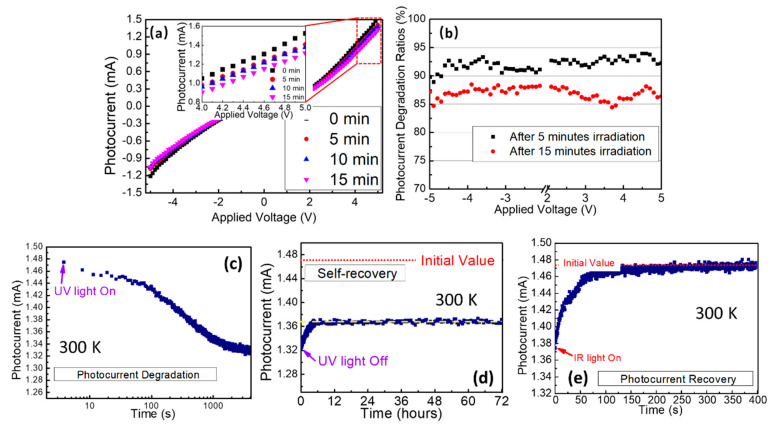
(**a**) Light-induced photocurrent degradation of the I–V response under different UV light exposure time (the inset is the enlarged photocurrent data in 4–5 V); (**b**) Light-induced photocurrent degradation rate versus applied bias voltage after 5- and 15-min irradiation; (**c**) Dynamics of the photocurrent intensity along with UV light exposure; (**d**) Dynamics of the self-recovered photocurrent intensity after UV light was turned off; (**e**) Dynamics of the recovered photocurrent intensity along with IR light exposure.

As seen in Figure 4a, light-induced degradation (LID) of the photocurrent response was observed when the device was exposed to UV light for a long time. After exposure to the UV light for 5 min, nearly 8% degradation of the photocurrent was measured, and it could reach 15% after 15 min exposure, as shown in Figure 4b. The dynamic curve of the photocurrent change after exposure to the UV light at 275 nm was also recorded by using the picoammeter, as seen in Figure 4c. The photocurrent decreased gradually with time along with the UV light exposure, and it reached the lowest value after half an hour of exposure. After turning off the UV light, the photocurrent dynamics were still recorded every 30 s. It was worth noting that the photocurrent could recover to some extent slowly in the dark circumstance itself, but it could not recover to its original value after several days, as seen in Figure 4d. When an infrared light at 808 nm was exposed on the device at this time, the photocurrent response could recover to its original value within several minutes, as seen in Figure 4e. However, it had nearly no photocurrent response when the device was exposed to the infrared light at this wavelength. 

**Figure 5:**
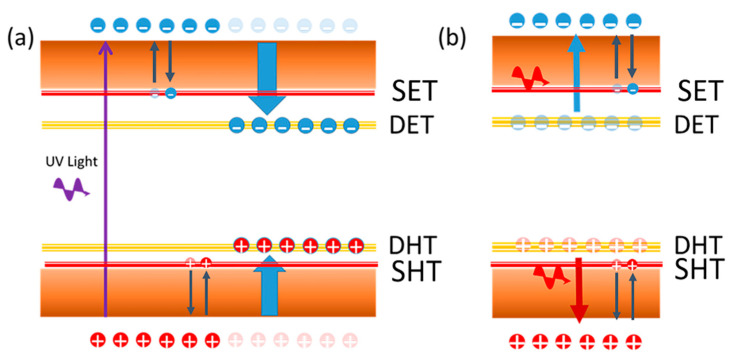
(**a**) Schematic illustration of the physical picture of deep electron traps and deep hole traps assisted light-induced degradation after UV light exposure; (**b**) Schematic illustration of the physical picture of the healing of light-induced degradation after infrared light exposure.

The deep defect centers caused by both V_Ga_ and V_N-_Mg_Ga_ vacancies played a vital role in the LID and healing processes of the photocurrent property above. As illustrated in Figure 5a, electrons on the valence band (VB) were excited into the conduction band (CB) when the UV light near the bandgap illuminated the surface of the photocurrent device. Then, the photocarriers could be detected by applying a bias voltage on the device. During this process, these photocarriers (both electrons and holes) could be captured by the abundant traps, including shallow traps and deep traps, at a certain probability. Recent research found that these photocarriers captured by shallow traps could escape from there by themselves at room temperature, and the degradation of photocurrent response could be healed by themselves [23,24]. However, the captured photocarriers in deep electron traps (DETs) and deep hole traps (DHTs) could not escape from the level of the trap at room temperature for a very long time. At this time, a part of the UV light source would be used to excite these trapped carriers from DET and DHT, followed by the degradation of the photocurrent response. As predicted by the defect photoluminescence spectra in Figure 2c, the energy level from DET and DHT to the valence band and conduction band could be estimated as 1.37–1.77 eV. As drawn in Figure 5b, when the infrared light at 808 nm was also exposed on the device, most of the captured carriers would be excited out of the traps because the photon energy matched the energy level well. The degradation behavior of the photocurrent recovered nearly to its original value rapidly, as shown in the dynamics of Figure 4e. The self-recovery of the photocurrent degradation in Figure 4d did not originate from the carriers released from the DET and DHT because these carriers cannot escape easily from there. Similar to the self-recovery of photocurrent degradation in halide lead perovskite, the self-recovery of the photocurrent was attributed to the self-release of the carriers at the shallow traps (ST). However, the number of these trapped carriers is very small, and the photocurrent performance that can be recovered over a long period of time is very limited at room temperature. Both V_Ga_ and V_N_-Mg_Ga_ defects exist in the Mg-doped GaN thin films, and Mg^2+^ ions doping concentration affected the number of the defects, which would also change the photocurrent degradation and recovery processes. To further investigate the LID and healing process, Hall coefficient and carrier concentration of the Mg-doped GaN thin film were measured by using the Hall measurement system with a magnetic field intensity of 600 mT. As listed in Table 2, a typical p-type conductivity thin film was obtained with a positive Hall coefficient of 9.33 cm^3^/C and a positive carrier concentration of 6.69 × 10^17^ /cm^3^. After exposing to the UV light for 5 min, the Hall coefficient increased to 14.17 cm^3^/C, and the hole concentration decreased to 4.40 × 10^17^ /cm^3^ since more photocarriers and free holes were captured by the DET and DHT. After that, the Hall coefficient and hole concentration recovered to 9.74 cm^3^/C and 6.37 × 10^17^ /cm^3^ under exposure to infrared light at 808 nm for 5 min.

Based on the experimental results and analysis above, DET and DHT contributed to the LID process in Mg-doped GaN thin films, which could be healed by exposure to the infrared light near the energy difference of the DET to the conduction band or DHT to the valence band. Apart from exposure to infrared light, the LID of the sample could also be healed by annealing the device under N_2_ atmosphere above 300 °C for more than 30 min. However, the testing device needs to be moved into the rapid thermal annealing system, and more defects might be introduced in the annealing process. The physical picture proposed in this work can be used in the application of light-induced degradation of photocurrent devices based on defects assisted electron and hole carrier traps, including in halide leads perovskites, zinc dioxide, and gallium nitrides semiconductors. Healing the LID to its initial state in a contactless way is desirable in integrated optoelectronic devices. 

## 4. Conclusions

To sum up, high-performance photocurrent devices with EQE of 1600% and responsivity of 4.7 A/W were investigated in Mg-doped GaN thin films, with a bandgap of 3.26 eV grown by using the MOCVD system. LID process of the photocurrent response was investigated when exposed to the UV light for a long time, which could be healed by illuminating to infrared light at 808 nm. Compared to the healing process by annealing at high temperature, recovery after exposure to the infrared light is contactless and will not introduce extra defects. This work provides a clear analysis of the light-induced degradation and the contactless healing of photocurrent devices based on defects assisted electron and hole carrier traps, including halide leads perovskites, zinc dioxide, and gallium nitrides semiconductors.

## Figures and Tables

**Table 1:** The growth parameters of the Mg doped GaN thin films.

	Growth Temperature	Flow Rate	Vacuum Pressure	Growth Time	Annealing Temperature
First Step	520 °C	TMG: 8.3 µmol/min NH_3_: 0.5 sccm	80 Torr	15 min	-
Second Step	850 °C	Cp_2_Mg: 0.4 µmol/min TMG: 8.3 µmol/min NH_3_: 0.5 sccm	80 Torr	120 min	-
Third Step	-	N_2_: 60 sccm	-	-	750 °C

**Table 2:** Hall coefficient and carrier concentration of the Mg-doped GaN thin film before and after UV or infrared light exposure.

Light Illumination	Magnetic Intensity	Hall Coefficient	Carrier Concentration
Before UV Illumination	600 mT	9.33 cm³/C	6.69 × 10^17^ cm^−3^
After UV Illumination for 5 min	600 mT	14.17 cm³/C	4.40 × 10^17^ cm^−3^
After Infrared light Illumination for 5 min	600 mT	9.74 cm³/C	6.38 × 10^17^ cm^−3^

## Data Availability

Not applicable.
